# ALK+ Anaplastic Large Cell Lymphoma (ALCL)-Derived Exosomes Carry ALK Signaling Proteins and Interact with Tumor Microenvironment

**DOI:** 10.3390/cancers14122939

**Published:** 2022-06-14

**Authors:** Dimitrios Chioureas, Janina Beck, George Baltatzis, Ioulia Vardaki, Pedro Fonseca, Nikolaos Tsesmetzis, Francisco Vega, Vasiliki Leventaki, Aristides G. Eliopoulos, Elias Drakos, George Z. Rassidakis, Theocharis Panaretakis

**Affiliations:** 1 Department of Oncology and Pathology, Karolinska Institutet, SE-17176 Stockholm, Sweden; dimitrios.chioureas@ki.se (D.C.); janina-beck@web.de (J.B.); gbaltatz@med.uoa.gr (G.B.); ioulia.vardaki@ki.se (I.V.); pedro.fonseca@ki.se (P.F.); nikolaos.tsesmetzis@ki.se (N.T.); tpanaretakis@mdanderson.org (T.P.); 2 Department of Hematopathology, The University of Texas MD Anderson Cancer Center, Houston, TX 77030, USA; fvega@mdanderson.org; 3 Department of Pathology, Children’s Hospital of Wisconsin & Medical College of Wisconsin, Milwaukee, WI 53226, USA; vleventaki@mcw.edu; 4 Department of Biology, School of Medicine, National and Kapodistrian University of Athens, 115 27 Athens, Greece; eliopag@med.uoa.gr; 5 Center of Basic Research, Biomedical Research Foundation of the Academy of Athens, 115 27 Athens, Greece; 6 Department of Pathology, University of Crete Medical School, 715 00 Heraklion, Greece; drakil@uoc.gr; 7 Department of Clinical Pathology and Cancer Diagnostics, Karolinska University Hospital, SE-17176 Stockholm, Sweden; 8 Department of Genitourinary Medical Oncology, The University of Texas MD Anderson Cancer Center, Houston, TX 77030, USA

**Keywords:** lymphoma, exosomes, microenvironment, NPM-ALK, cytokines

## Abstract

**Simple Summary:**

ALK+ anaplastic large cell lymphoma (ALK+ ALCL) is a distinct type of aggressive non-Hodgkin lymphoma of T-cell origin, which is characterized by overexpression and activation of ALK kinase due to chromosomal translocations of the gene. The most frequent chromosomal aberration is the t(2;5) resulting in the NPM-ALK chimeric protein, which exerts its oncogenic functions through activation of multiple oncogenic pathways. Exosomes, the best characterized type of extracellular vesicles, are secreted from the tumor cells, thus transferring signals to other cells that uptake exosomes. In this study, we demonstrate that ALK+ ALCL cells secrete exosomes that carry critical molecules of ALK signaling, which can be taken up by other cells with significant biologic effects including functional interactions with tumor microenvironment cells, which may contribute to tumor aggressiveness and possibly resistance to treatment.

**Abstract:**

The oncogenic pathways activated by the NPM-ALK chimeric kinase of ALK+ anaplastic large cell lymphoma (ALCL) are well characterized; however, the potential interactions of ALK signaling with the microenvironment are not yet known. Here we report that ALK+ ALCL-derived exosomes contain critical components of ALK signaling as well as CD30, and that exosome uptake by lymphoid cells led to increased proliferation and expression of critical antiapoptotic proteins by the recipient cells. The bone marrow fibroblasts highly uptake ALK+ ALCL-derived exosomes and acquire a cancer-associated fibroblast (CAF) phenotype. Moreover, exosome-mediated activation of stromal cells altered the cytokine profile of the microenvironment. These interactions may contribute to tumor aggressiveness and possibly resistance to treatment.

## 1. Introduction

Anaplastic lymphoma kinase-positive (ALK+) anaplastic large cell lymphoma (ALCL) is a distinct T-cell lymphoma type characterized by overexpression and activation of ALK due to chromosomal translocations of *ALK* gene locus at 2p23 [1]. The most common translocation is the t(2;5)(p23;q35)2 resulting in the chimeric NPM-ALK, which activates multiple oncogenic pathways including the Ras/ERK, JAK/STAT3, PI3K/AKT/MTOR, JNK/Jun, Sonic Hedgehog and others [2]. ALK+ ALCL is also characterized by expression of CD30 receptor, a common feature for both ALK+ and ALK- ALCL.

The role of the tumor microenvironment (TME) in lymphoma progression and drug resistance has been recognized over the past few years. TME is characterized by stromal cells such as fibroblasts, mesenchymal stem cells, follicular dendritic cells, and inflammatory cells such as macrophages, T- and B-lymphocytes. Various components of the TME may interact with the tumor cell, thus contributing to lymphoma cell survival and proliferation. The cross-talk between the neoplastic cells and the TME is mediated by autocrine as well as paracrine signaling such as cytokines, growth factors and secreted extracellular vesicles. Exosomes, the best characterized type of extracellular vesicles, represent the newest family of bioactive vesicles that originate from the endosome and are actively secreted by virtually all cell types under normal as well as pathological conditions [3]. Exosomes range between 30–100 nm in diameter, and contain a set of characteristic exosomal proteins (e.g., tetraspanins) as well as molecules (proteins, coding and non-coding RNA and DNA) specific for the cell type of origin. In certain hematologic malignancies such as chronic lymphocytic leukemia, exosomes play a pivotal role in shaping the TME in favor of tumor growth [4]. However, the molecular content and functional properties of ALCL-derived exosomes are not yet known.

## 2. Results and Discussion

### 2.1. Molecular Characterization of ALK+ ALCL-Derived Exosomes

**Figure 1:**
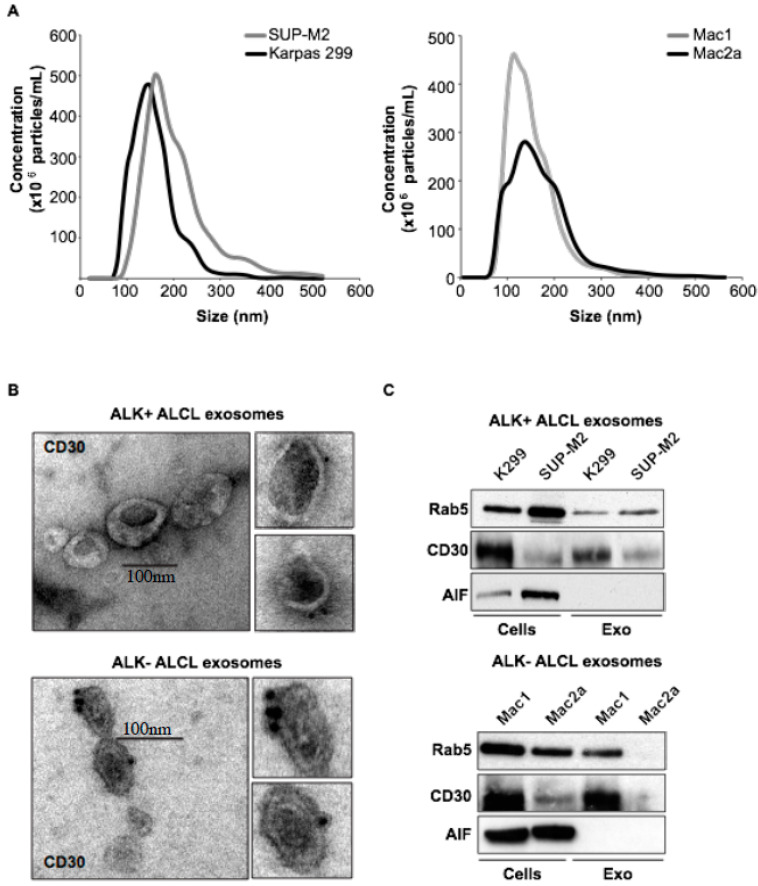

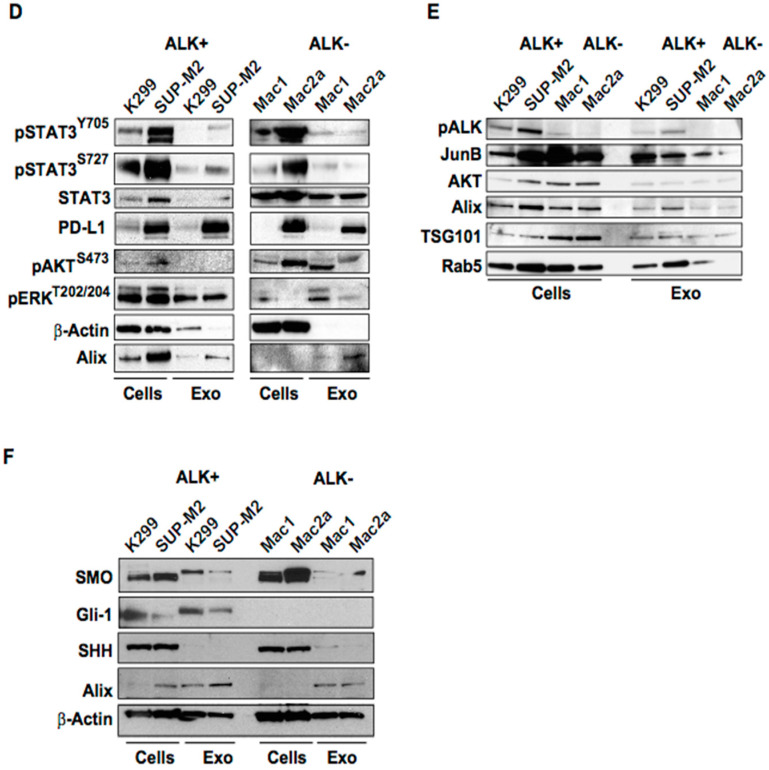
**Molecular profiling of ALK+ and ALK- ALCL-derived exosomes:** (**A**) Nanoparticle tracking analysis (NTA) for the ALK+ (Karpas 299, Sup-M2) and ALK- (Mac1, Mac2A) ALCL exosomes. The mean size and particle concentration of the preparations are shown; (**B**) Immuno-electron microscopy (iEM) staining with gold particles for CD30 on the ALCL-derived exosomes; (**C**) Western blot analysis of the ALK+ (upper panel) and ALK- (lower panel) ALCL whole cell and exosomal lysates shows the protein levels of Rab5 (exosomal marker), CD30, and AIF (quality control for the exosomal preparations); (**D**) Protein composition of the ALK+ and ALK- cell lines and exosomes. Alix is an exosomal marker; (**E**) Alongside the ALK+ ALCL cells and their respective exosomes are the NPM-ALK oncoprotein. The AP-1 transcription factor, known to be unregulated in ALK+ ALCL by NPM-ALK, is detected at a higher level in exosomes derived from ALK+ than ALK- ALCL cells. 11 Alix, Tsg101 and Rab5 are characteristic exosomal proteins; (**F**) Two crucial components of the sonic hedgehog pathway, SMO and Gli-1, which are known to be upregulated by NPM-ALK, are detected at a higher level in exosomes secreted by ALK+ than ALK- ALCL cell lines. Original images of western blot can be found at File S1 and donsitometry data for Western blots are shown in Figure S1.

Here we analyze for the first time the molecular content of the ALK+ and ALK- ALCL-derived exosomes using an in vitro model of ALK+ (Karpas 299, SUP-M2) and ALK- (Mac1, Mac2) ALCL. Exosomes were isolated using ultracentrifugation protocols. Abundant exosome secretion was seen in both ALK+ and ALK- ALCL cell lines by Nanoparticle Tracking Analysis (NTA), which measures the size and the relative particle concentration, and by transmission electron microscopy (Figure 1A,B). CD30 receptor was detected in the ALCL-derived exosomes by immuno-electron microscopy gold nanoparticle staining and Western blot analysis (Figure 1C). Hansen and co-workers have demonstrated a CD30 vesicle-containing network in lymphoid tissue of classical Hodgkin lymphoma (cHL), which might facilitate the communication between distant cell types in cHL tissue and allow a functional CD30–CD30L interaction in trans [5]. Better understanding of the potential role of CD30 in the tumor cell microenvironment may have therapeutic implications in the era of CD30-targeted therapy.

In this study, we also found, for the first time, that activated ALK and critical components of the ALK signaling are present in the exosomes secreted by ALK+ ALCL cells indicating activation of Ras/ERK, JAK/STAT3, PI3K/AKT/mTOR and Sonic Hedgehog pathways (Figure 1D–F). The whole cell and exosomal lysates were probed for known exosomal markers as well as for key proteins of the ALK oncogenic pathway. Both ALK+ and ALK-derived exosomes bear proteins of the activated oncogenic pathways such as pSTAT3Y705, pAktS473 and pERKT202/204. Of note, JunB [6], known to be upregulated by NPM-ALK, was enriched at a higher level in ALK+ compared to ALK- ALCL exosomes. Activated STAT3 and AKT, which represent common signaling components in both ALK+ and ALK- ALCL are detected at a variable level in all ALCL-derived exosomes (Figure 1D–F). Interestingly, PD-L1, a crucial regulator of the immune checkpoint, which is differentially expressed among the ALK+ and the ALK- ALCL cell lines and tumors [7] is present in the exosomes (Figure 1D–F). Whether exosomal PD-L1 functionally interacts with the PD-1 on the T cells in TME and may affect the response to immune checkpoint immunotherapy certainly merits further investigation. Mac2A cells expressed higher levels of exosomal PD-L1 than Mac1, and, notably, Mac2A were derived from the same patient (as Mac1) cells at a later, more advanced stage of disease. To the best of our knowledge, detection of PD-L1 has not been demonstrated in any lymphoma-derived exosomes to date.

### 2.2. ALCL-Derived Exosomes Can Be Taken up by Lymphoid Cells

**Figure 2:**
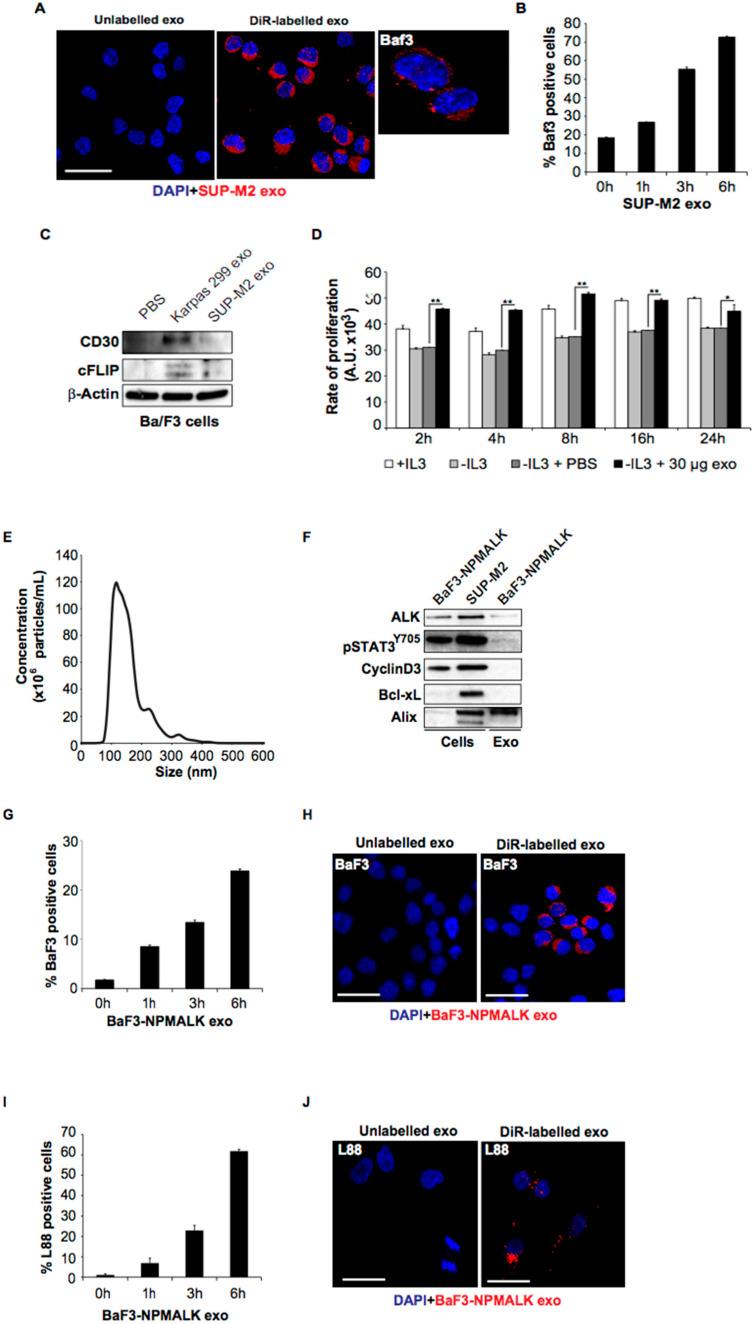
**ALK+ ALCL-derived exosomes are taken up by Ba/F3 cells with apparent biologic effects:** (**A**,**B**) DiR-labelled SUP-M2 secreted exosomes (20 μg/mL) were added to the Ba/F3 cells for the time points as indicated and the uptake was quantified by flow cytometry and depicted by confocal microscopy. After 6 h, the exosome uptake reached a level of approximately 75% as shown (**B**); (**C**) Immunoblots showing detection of CD30 and the anti-apoptotic protein cFLIP in Ba/F3 cells following uptake of exosomes derived from two ALK+ ALCL cell lines, Karpas 299 and SUPM2; (**D**) Proliferation of Ba/F3-cells with or without IL-3 (10 ng/mL) and 30 µg SUP-M2-exosomes. 10^4^ Ba/F3-cells in a volume of 100 µL were cultured in the settings as shown for 48 h. To determine the proliferation of the cells, 20 µL CellTiter-Blue^®^ Reagent (Promega, Madison, WI, USA) was added and the fluorescence at 560/590 nm was determined at the indicated timepoints. The highest level of proliferation fraction in the cells treated with 30 µg SUP-M2 exosomes (no IL-3) was observed at 8 h and exceeded that of IL-3 treated cells (* *p* < 0.05, ** *p* < 0.01); (**E**) Nanoparticle tracking analysis (NTA) for the Ba/F3-NPM-ALK-derived exosomes; (**F**) The immunoblots (Western blot) show total ALK, pSTAT3^Y705^, CyclinD3, Bcl-xL and Alix protein levels for the Ba/F3-NPM-ALK cells and exosomes compared to the SUP-M2 cells. The Ba/F3-NPM-ALK is a Ba/F3 clone stably transfected with NPM-ALK construct as described in Materials and Methods section. Alix was used as an exosomal marker; (**G**,**H**) DiR-labelled Ba/F3-NPM-ALK exosomes (20 μg/mL) were added to the Ba/F3 cells for the time points as indicated and the exosome uptake was quantified by flow cytometry (**G**) and depicted by confocal microscopy (**H**). At 6 h, the uptake reached a level of 25% as shown; (**I**,**J**) DiR-labelled Ba/F3-NPM-ALK exosomes (20 μg/mL) were added to the L88 bone marrow-derived fibroblasts for the time points as indicated and the exosome uptake was quantified by flow cytometry (**G**) and depicted by confocal microscopy (**H**). At 6 h, the uptake exceeded a level of 60% as shown. Original magnificationn for A, H, J: X1000. Original images of western blot can be found at File S1 and donsitometry data for Western blots are shown in Figure S1.

The ALCL-derived exosomes were readily taken up by various cell types including murine pro-B lymphoid cells, Ba/F3 (Figure 2A,B). The uptake was time-dependent and reached a level of 75% at 6 h. Proteins present in the exosomes such as the CD30 receptor were detected in Ba/F3 cells following exosome uptake and, furthermore, the recipient cells acquired anti-apoptotic properties such expression of cFLIP, which has been previously shown to confer resistance to FAS-mediated apoptosis in ALCL (Figure 2C) [8]. Notably, proliferation of Ba/F3 cells was increased in the presence of ALCL-derived exosomes with the highest level being observed at 8 h, and at this point the proliferation fraction exceeded that of IL-3-treated cells (Figure 2D). Taken together, these findings suggest that the ALK+ ALCL-derived exosomes are enriched in oncogenic signals, and their uptake by recipient cells leads to anti-apoptotic and cell proliferation effects. Export of oncogenic signaling by exosomes with functional properties has been described in aggressive B-cell lymphomas as well [9]. Moreover, we show that exosomes secreted by the Ba/F3 cell line stably transfected with NPM-ALK are potently taken up by paternal Ba/F3 cells in a time dependent manner (Figure 2E–J). These findings provide evidence of functional exosome uptake by non-neoplastic lymphoid cells (Ba/F3) as well. Expression of pALK and pSTAT3Y705 confirmed the presence of an active NPM-ALK kinase in Ba/F3-NPM-ALK cells. The activated (phosphorylated) form of STAT3, pSTAT3Y705, but neither the cell cycle regulator Cyclin D3 nor Bcl-xL were detected in the exosomes derived from Ba/F3-NPM-ALK.

### 2.3. ALCL-Derived Exosomes Interact with the Tumor Microenvironment Cells

**Figure 3:**
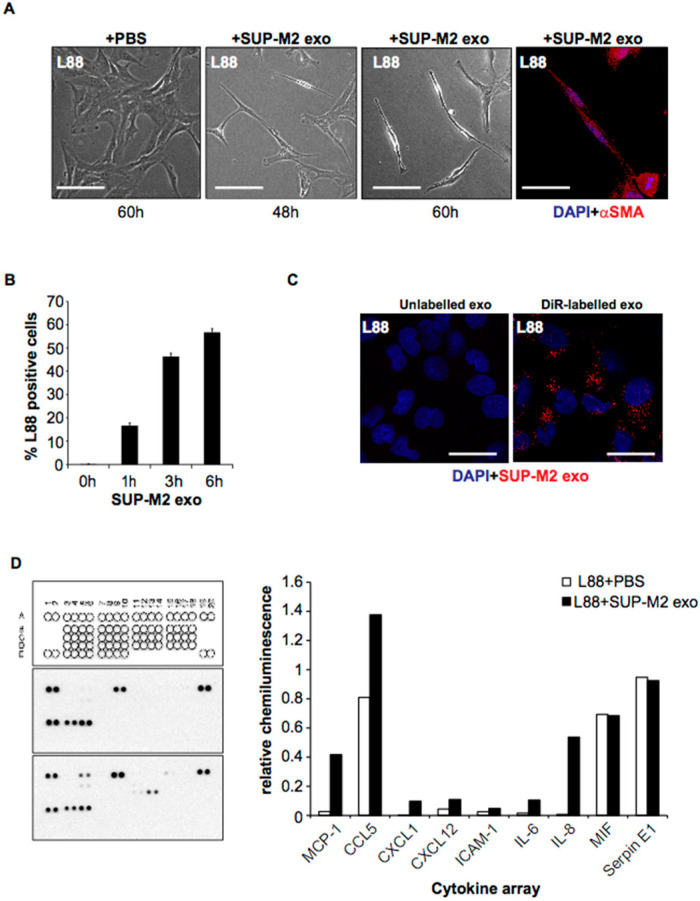

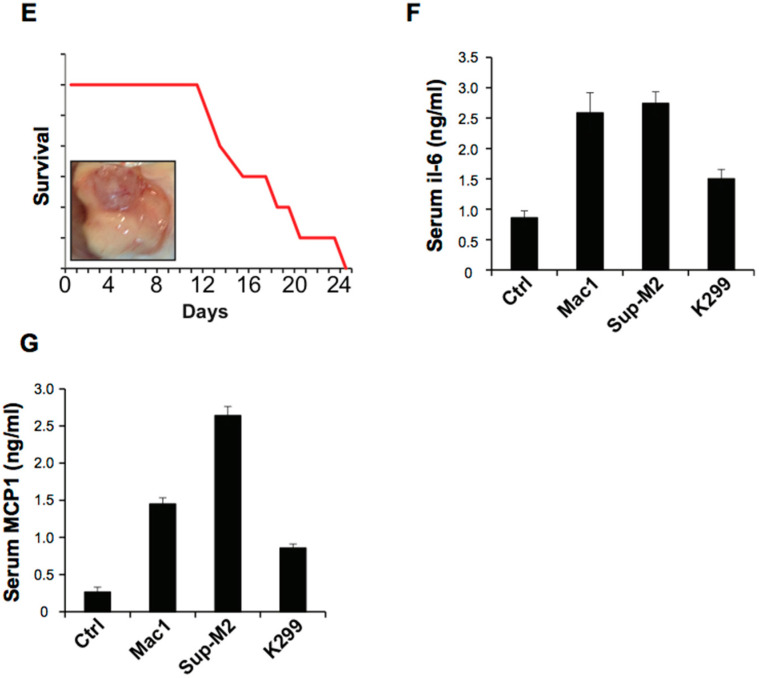
**Effects of ALK+ ALCL-derived exosomes on the bone-marrow-derived fibroblasts and cytokine profile:** (**A**,**B**) L88 cells were educated with 60 μg/mL of SUPM2-derived and DiR-labelled exosomes at the indicated time points. The positive population was quantified by flow cytometry (**A**) and depicted by confocal microscopy (**B**) after 6 h of uptake; (**C**) Morphological changes of bone marrow-derived L88 fibroblasts due to education with SUPM2-derived exosomes (60 µg/mL) for 60 h. Incubation of the L88 cells with ALCL-derived exosomes also confers molecular changes such as expression of aSMA in the fibroblasts characteristic of the transformation to a cancer-associated fibroblast (CAF) phenotype; (**D**) The cells’ supernatant was recovered and used for the cytokine array assay as described in Materials and Methods. The cytokine expression was captured on X-ray films (left panel) and the differences were visualized by bar charts after normalization (white bars for PBS/control and black bars for SUP-M2 exosomes). (**E**) 10^7^ cells from two ALK+ ALCL cell lines, Karpas 299 and SUPM2, and one ALK- ALCL cell line, Mac1, were injected subcutaneously in SCID/beige mice (10 mice for each cell line) and were followed for tumor development. The tumors were measured and the mice were sacrificed when the tumor reached 1.5 cm in max. diameter according to the institutional ethical guidelines. A representative example of SUPM2 xenografts and the survival of this group of SCID/beige mice was calculated (Kaplan–Meier curve); (**F**,**G**) Plasma was isolated from all SCID/beige mice. Plasma samples were thawed on ice, briefly mixed and spun down at 500 g for 30 s. Supernatant was collected in a new tube, diluted 1:1 with ice-cold PBS 1× and spun down at 12,000× *g* for 45 min at 4 °C. Supernatant was collected in a new tube and kept on ice until use (100 μL per well). IL-6 and MCP1/CCL2 levels were assessed with ELISA, which was performed according to the manufacturer’s recommended protocol (Mini ELISA Development kits, PeproTech Nordic, Stockhol, Sweden). Results were obtained by using Spark 10M plate reader (Tecan, Männedorf, Switzerland).

To investigate the role of ALCL-derived exosomes on the tumor stroma, we examined the uptake of ALCL-derived exosomes by the bone marrow stroma-derived L88 fibroblasts. We found that incubation of the L88 cells with ALCL-derived exosomes confers morphological and molecular changes (expression of aSMA) in the fibroblasts that undergo transformation to cancer-associated fibroblasts (CAF) (Figure 3A–C). It is well-established that the tumor cells induce CAF phenotype, and the activated fibroblasts, in turn, produce a number of growth factors and cytokines that promote, among other cellular functions, cell proliferation and angiogenesis. 

Having shown the functional interaction of ALCL-secreted exosomes with L88 fibroblasts, we sought to investigate whether exosome-mediated activation of stromal cells altered the cytokine profile of the TME. Using a cytokine array, the level of certain cytokines including MCP-1/CCL2, CCL5/RANTES, IL-6 and IL-8 was substantially increased in the medium following incubation with ALK+ ALCL-secreted exosomes (Figure 3d) with the highest relative increase being observed for IL-8 and MCP-1/CCL2. Notably, MCP-1/CCL2 and IL-6 levels were found to be elevated in the plasma of SCID/beige mice injected with two ALK+ (SUPM2, Karpas 299) and one ALK- (Mac1) ALCL cell lines (Figure 3E–G). 

In a previous study, Dejean and co-workers [10] showed that high-mobility-group box-1 (HMGB-1), a proinflammatory cytokine, is released by ALK+ ALCL cells, and extracellular HMGB-1 stimulated secretion of the IL-8 chemokine by other cell types as epithelial cells of the skin (keratinocytes). IL-8 was capable of inducing invasiveness and dissemination of ALK+ ALCL cells. This is of particular interest, since ALK+ ALCL express the IL-8 receptors CXCR1 and CXCR2. Therefore, it is tempting to speculate that ALK+ ALCL-secreted exosomes induce IL-8 production by the CAFs, which are secreted in the microenvironment and IL-8, in turn, interacts with the tumor cells bearing the IL-8 receptors. The role of monocyte chemoattractant protein-1/CCL2 (MCP-1/CCL2) in ALCL pathogenesis and progression is not yet known. The MCP-1/CCL2 and its receptor CCR2 regulate migration from the blood across the vascular endothelium and tissue infiltration of monocytes and macrophages, which play a significant role in immunological surveillance and inflammation. 

Taken together, we have demonstrated that ALK+ ALCL cells secrete exosomes that bear critical molecules of ALK signaling [11,12,13,14,15,16,17,18,19,20], which can be taken up by lymphoid cells with apparent biologic effects. Functional interactions of the ALK+ ALCL cells with bone marrow stroma cells lead to fibroblast activation (CAFs) and alter the cytokine profile of the TME, which may contribute to tumor aggressiveness and possibly resistance to treatment.

## 3. Materials and Methods

### 3.1. Cell Lines and Culturing Conditions

The details of the cell lines used in the study are provided in Supplementary Table S1. Karpas 299 and Sup-M2 (ALK+ ALCL), Mac1 and Mac2A (ALK- ALCL), L88 (bone marrow derived fibroblasts) cell lines were cultured in 175 cm^2^ flasks in RPMI 1640 (Hyclone, GE Healthcare Life Sciences, Logan, UT, USA) supplemented with 10% fetal bovine serum (Hyclone, GE Healthcare Life Sciences), glutamine (2 mM), penicillin and streptomycin (50 μg/mL) (Life Technologies, Carlsbad, CA, USA) and incubated at 37 °C in a humidified atmosphere containing 5% CO_2_. Ba/F3 (pro-B murine lymphoid cells) were cultivated at the same conditions with the addition of recombinant IL-3 (10 ng/mL, Peprotech). 

### 3.2. Exosome Isolation

In order to deplete the medium from the FBS-derived exosomes, RPMI 1640 enriched with 30% FBS was centrifuged overnight at 100,000× *g*, at 4 °C. Afterwards, it was filtered in 0.22 μm disposable filters and diluted to 10% FBS for cell culturing. The ALCL cell lines were cultured in 175 cm^2^ flasks at the density of 0.6 × 10^6^ cells per ml for 48 to 72 h. Then, the cells were separated from the supernatants by centrifugation at 100× *g*, RT, and the collected supernatants were filtered in 0.22 disposable filters. The filtered supernatant was centrifuged for 2 h at 100,000× *g*, at 4 °C. The supernatant was discarded and the pellet was washed with PBS and centrifuged for 2 h at 120,000× *g*, at 4°C. The pellet was resuspended in PBS and stored in aliquots at −80 °C.

### 3.3. Nanoparticle Tracking Analysis (NTA)

Nanoparticle tracking analysis (NTA) was performed to determine the size and particle number of the exosomal preparation using the LM10 (NanoSight Limited, London, UK) equipped with an 8 mega pixel camera (Andor Technology, Tokyo, Japan). A 405 nm laser was used for the detection of the exosomes and the NTA software for data acquisition and analysis. Five different videos were used for capturing for 60 s and the videos were analyzed with camera level set at 14 and detection threshold of 15.

### 3.4. Western Blot Analysis

The cell pellets and exosomes were lysed in RIPA lysis buffer (10 mM Tris, pH 7.2, 150 mM NaCl, 1% deoxycholate, 1% Triton, 0.1% SDS, 5 mM EDTA) containing complete protease inhibitor cocktail, phospho-stop (Roche Diagnostics, Meylan, France), dithiothreitol (Sigma Aldrich, St. Louis, MO, USA) and vanadate (Life technologies, Carlsbad, CA, USA). Equal amounts of soluble protein (30 micrograms) were loaded in precast SDS-PAGE gels (Life technologies, Carlsbad, CA, USA) and transferred to PVDF membranes for probing with specific primary antibodies overnight at 4 °C and subsequently with horseradish peroxidase-conjugated secondary antibody. Detection of the protein bands was achieved using chemiluminescence (Life technologies, Carlsbad, CA, USA) and exposure on X-ray films (Kodak Rochester, NY, USA). The t-test was used for the comparisons of various measurements between control and experimental conditions as well as between densitometric data in Western blots. *p* value < 0.05 was considered statistically significant.

### 3.5. DiR Labelling

The exosomes were labelled with DiR fluorescent dye as previously described. Briefly, 0.5 mM fluorescent lipophilic tracer DiR was added to the cells’ supernatant and incubated for 15 min at room temperature before centrifugation, as mentioned above. After centrifugation, the supernatant was kept and used as control for the relevant experiments.

### 3.6. Exosome Uptake Assays

For the uptake assays, the amounts of exosomes used were determined by the Pierce BCA protein assay (Life technologies, Carlsbad, CA, USA). The cells were plated in 24-well plates in serum free medium overnight before the addition of the exosomes. The positive population was recorded by flow cytometry (FACS LSRII, BD, Franklin Lakes, NJ, USA) and the analysis was performed with the FACS DIVA software (BD, Franklin Lakes, NJ, USA). For imaging, the cells were grown on coverslips (adherent cells) or cytospun, fixed in 4% PFA and mounted in mounting medium with DAPI (VECTOR LABORATORIES, Burlingame, CA, USA). Images were captured with a Leica confocal microscope.

### 3.7. Cytokine Array

The Human Cytokine Array Kit (R&D Systems, Minneapolis, MN, USA) was used according to the manufacturer’s instructions. The exposure was performed on X-ray films (Kodak, Rochester, NY, USA). The band density was quantified with the ImageJ software (NIH, Bethesda, MD, USA). The mean values of the duplicates were subtracted by the negative control and normalized with the positive control. The analysis and depiction were performed by Excel software (Microsoft, Redmond, WA, USA).

### 3.8. Cell Proliferation Assays

For the proliferation assays, BaF3 cells were cultivated in serum- and IL-3- free RPMI 1640-medium for 24 h. The cells were plated in 96-well plates in a volume of 100 µL/well. PBS, IL-3 (10 ng/mL, Peprotech) and Sup-M2 exosomes were added. After 48 h incubation, 20 µL CellTiter-Blue^®^ Reagent (Promega, Madison, WI, USA) was supplemented. Fluorescence was measured at a wavelength of 560/590 nm with a Tecan Sparks 10M (Tecan Group Ltd., Männedorf, Switzerland) after 2, 4, 8, 16 and 24 h incubation. The analysis was carried out with Excel software (Microsoft, Redmond, WA, USA).

### 3.9. Electron Microscopy

For immunogold labeling for electron microscopy, exosomes were prepared and labeled according to previous described protocols. In brief, isolated exosomes were resuspended in 2% paraformaldehyde (PFA), adsorbed unto nickel Formvar-carbon coated electron microscopy grids (200 mesh), blocked with PBS/5% (*w*/*v*) BSA and incubated with CD30 antibody (DAKO, A/S, Denmark) for 30 min. Subsequently, grids were washed, blocked and incubated with Donkey Anti-Mouse IgG H&L 10 nm Gold antibody (Abcam, Cambridge, United Kingdom). Grids were washed, fixed with 1% glutaraldehyde, contrasted with 4% uranyl acetate and finally embedded in a mixture of 4% uranyl acetate and 2% methyl cellulose. Grids were visualized on a Morgagni 268 Electron Microscope (FEI, Eindhoven, The Netherlands) and photographed with the Morada Soft Imaging System (Olympus Corporation, Hamburg, Germany).

### 3.10. Molecular Cloning of NPM1-ALK Full Length Fusion

The full-length cDNA sequences (2043 bp) of *NPM1-ALK* were amplified from a previously cloned pcDNA-DEST40 Gateway vector (Invitrogen) containing the cDNA sequence of *NPM1-ALK*, using the primers: forward, 5′-CGCGGTATAGCGGCCGCCATGGAAGATTCGATGGACATGGACA-3′; reverse, 5′-GAGAGGGGCGGAATTCTCAGGGCCCAGGCTGGTTCAT-3′. The PCR fragments were purified using QIAquick PCR Purification Kit (Qiagen) and then cloned into the MSCV-IRES-GFP mammalian expressing retroviral vector (St. Jude Children’s Research Hospital Vector Core, Memphis, TN, USA) between *Not1* and *EcoR1* restriction sites, using In-Fusion cloning kit (Clontech). The In-Fusion reaction was performed in a total volume of 10 µL, containing 2.0 µL of 5× In-Fusion HD Enzyme Premix, 200 ng of each purified PCR fragment, 200 ng of the retroviral vector and dH_2_O from the In-Fusion HD Cloning Kit (Clontech). The reaction mix was incubated at 50 °C for 15 min, and then placed on ice for transformation using *E. coli* HST04 Premium Competent Cells (Clontech). The full-length cDNA sequences (2043 bp) of *NPM1-ALK* in infectious viral clones were determined by Sanger sequencing. 

### 3.11. Retroviral Construct and Infection

The murine hematopoietic progenitor Ba/F3 cell line was purchased from ATCC (Manassas, VA, USA). Cells were cultured in RPMI-1640 medium containing 25 mM Hepes (ThemoFisher Scientific) supplemented with 10% heat-inactivated fetal bovine serum (HyClone), 2 mmol/L L-glutamine, 100 U/mL penicillin, 100 µg/mL streptomycin and 10 ng/mL IL-3 (PeproTech, Cranbury, NJ, USA). For all experiments, cells were used during their exponential growth phase. Viral-producing supernatants containing MSCV-NPM-ALK-IRES-GFP were produced using the ecotropic Phoenix packaging cell line and supernatants harvested 72 h after transfection were used to infect Ba/F3 cells. 

### 3.12. Human Tumor Xenografts

Animals were housed under pathogen-free conditions, fed autoclaved standard chow and water ad libitum. Ketamine and xylazine (each 0.45 mg/mouse; Sigma-Aldrich, Munich, Germany), administered intraperitoneally, were used for anesthesia. Decreased activity, ill thrift, anorexia, dyspnea, or tumor diameter greater than 1.5 cm were used as criteria for sacrifice. Animal care and use was in accordance with guidelines of the Foundation for Research and Technology, Athens, Greece. Animal experiments were approved by the University of Crete ethics committee (protocol code: 2181/15-04-2016).

For the in vivo studies we used SCID/Beige female mice. 10^7^ cells of the ALK+ (Karpas 299, Sup-M2) and ALK- (Mac1) ALCL cell lines were diluted in 100 μL of HBSS without calcium, magnesium or phenol red (Life technologies, Carlsbad, CA, USA) and injected subcutaneously in the thigh of the mice. When the growing tumor was palpable, it was measured every day with a caliper. The volume size was calculated with the formula V = width × height × depth/2. When the mice were sacrificed, the blood was extracted from the heart and the plasma was cleaned and isolated with differential centrifugations and stored in −80 °C for further experiments.

### 3.13. ELISA Assays

The levels of the expressed IL-6 and CCL2/MCP-1 cytokines in the bloodstream of the mice were measured with Murine IL-6 Standard ABTS ELISA Development Kit and Murine JE/MCP-1 Standard ABTS ELISA Development Kit (Peprotech) according to the manufacturer’s instructions. The detection was performed in a 150 Tecan Sparks 10M (Tecan Group Ltd., Männedorf, Switzerland) plate reader set at 405 nm with wavelength correction set at 650 nm.

## 4. Conclusions

We have shown that ALK+ ALCL cells secrete exosomes that bear critical molecules of ALK signaling, which can be taken up by lymphoid cells with apparent biologic effects. Functional interactions of the ALK+ ALCL cells with bone marrow stroma cells lead to fibroblast activation (CAFs) and alter the cytokine profile of the TME, which may contribute to tumor aggressiveness and possibly resistance to treatment.

## Data Availability

The data presented in this study are available in this article and Supplementary Material.

## Supplementary Materials

The following are available online at https://www.mdpi.com/article/10.3390/cancers14122939/s1, File S1: WB original images. Table S1: List of cell lines used in the present study. Figure S1: Donsitometry data for Western blots are shown below for all relevant figures
