# Supplementary material for: ALK+ Anaplastic Large Cell Lymphoma (ALCL)-Derived Exosomes Carry ALK Signaling Proteins and Interact with Tumor Microenvironment
Source: Cancers (Basel). 2022 Jun 14;14(12):2939. doi: 10.3390/cancers14122939 (PMC9221431; doi:10.3390/cancers14122939)
Supplement: Supplementary file 1 [file cancers-14-02939-s001.zip › cancers-1672707-FileS1.pdf]

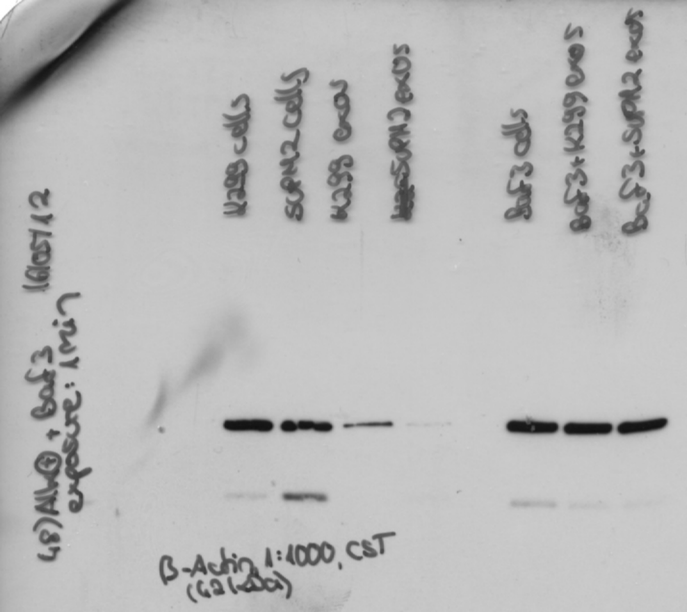
K299 cells
SUPM2 cells
K299 exos
SUPM2 exos
BaF3 cells
BaF3+K299 exos
BaF3+SUPM2 exos
48) ALK+ + BaF3
exposure: 1min
β-Actin 1:1000, CST
(42kDa)

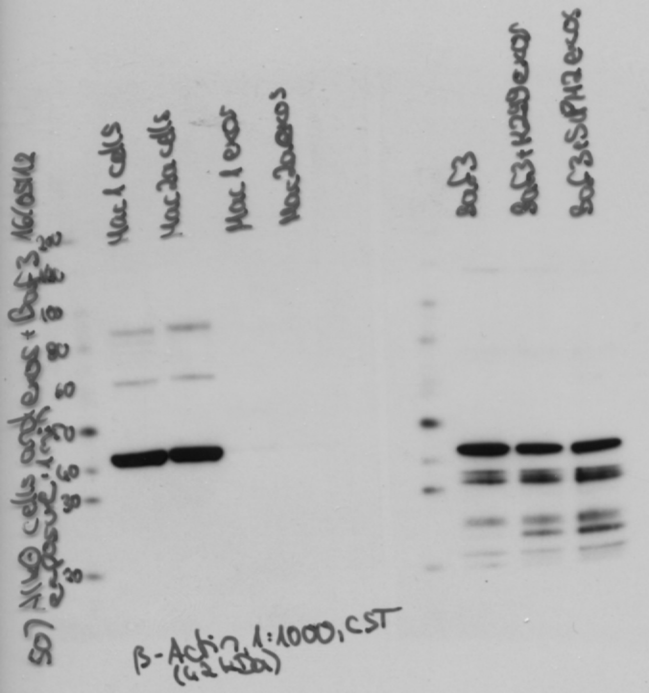
Mac 1 cells
Mac 2a cells
Mac 1 exos
Mac 2a exos
BaF3
β-Actin 1:1000, CST
(42 kDa)

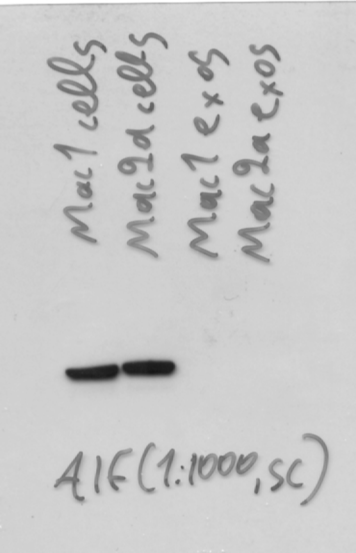
Mac1 cells
Mac2a cells
Mac1 exos
Mac2a exos
AIF (1:1000, sc)

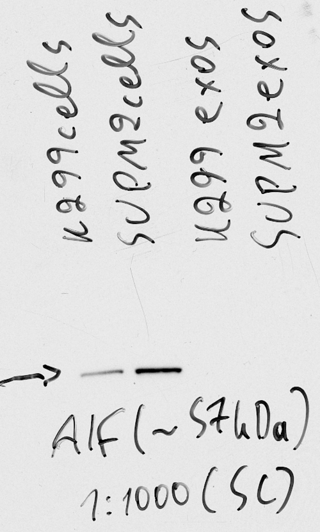
K299cells
SUPM2cells
K299 exos
SUPM2exos
AIF (~57kDa)
1:1000 (SC)

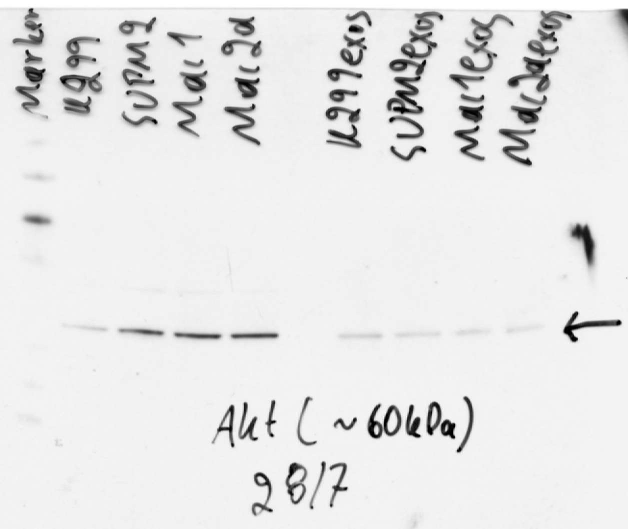
Marker
K299
SUPM2
Mac1
Mac2A
K299exos
SUPM2exos
Mac1exos
Mac2Aexos
Akt (~60kDa)
28/7

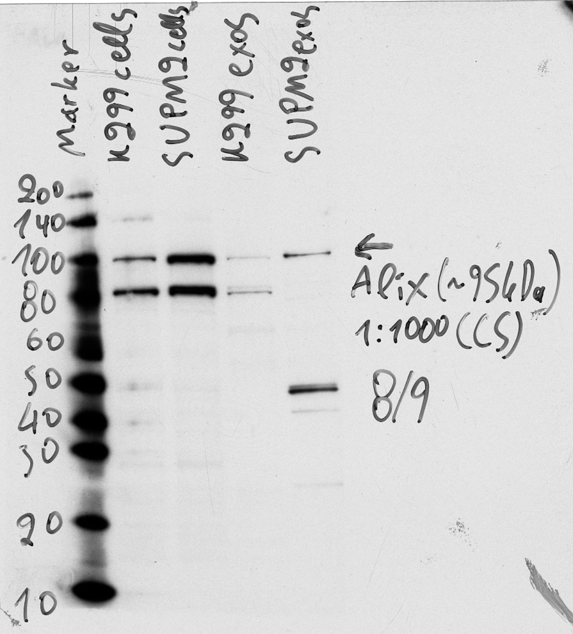
Marker
K299 cells
SUPM2 cells
K299 exos
SUPM2 exos
200
140
100
80
60
50
40
30
20
10
Alix (~95kDa)
1:1000 (CS)
8/9

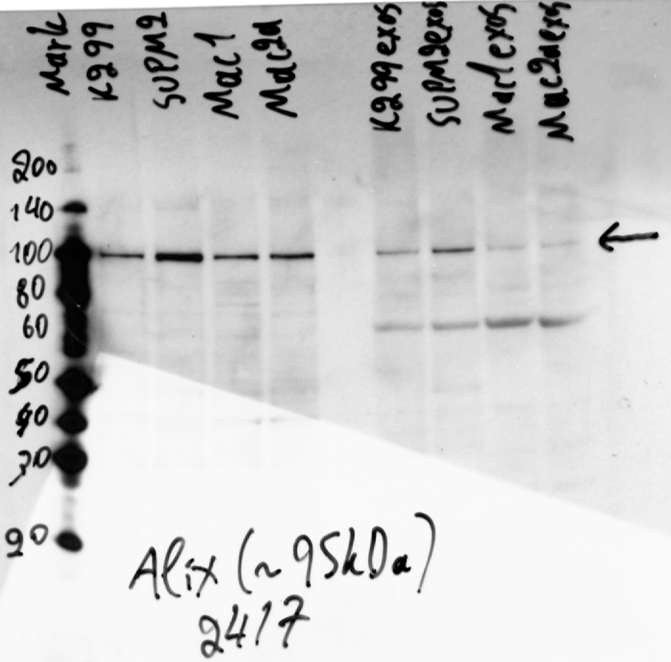
Mark
K299
SUPM2
Mac1
Mac2a
K299exos
SUPM2exos
Mac1exos
Mac2aexos
200
140
100
80
60
50
40
30
20
Alix (~95kDa)
24/7

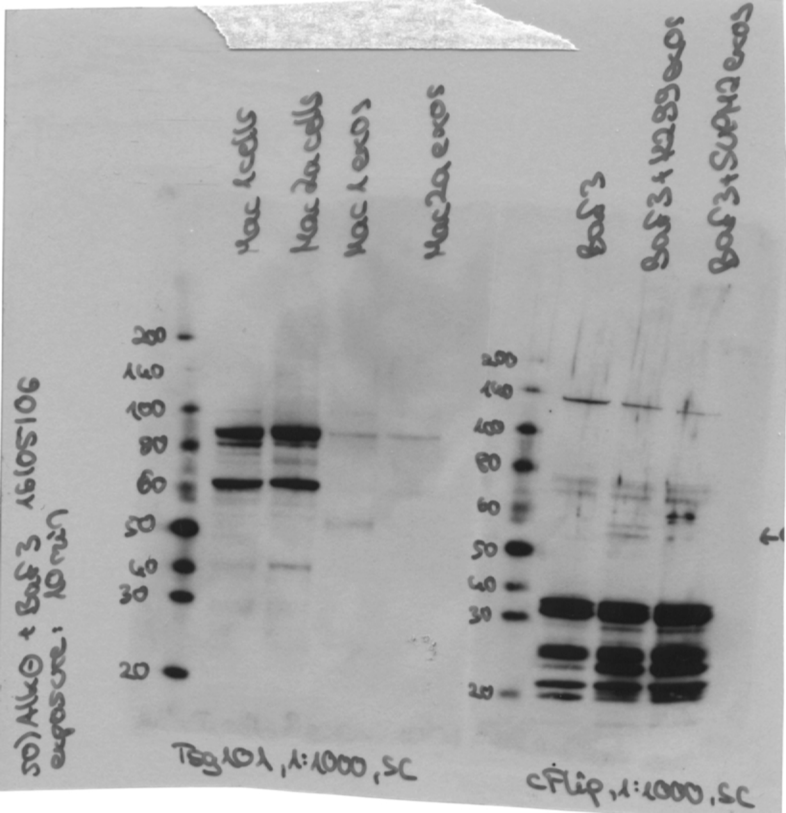

Mac1 cells
Mac2a cells
Mac1 exos
Mac2a exos
BaF3
200
140
100
80
60
50
40
30
20
exposure: 10 min
Tsg101, 1:1000, SC
cFlip, 1:1000, SC

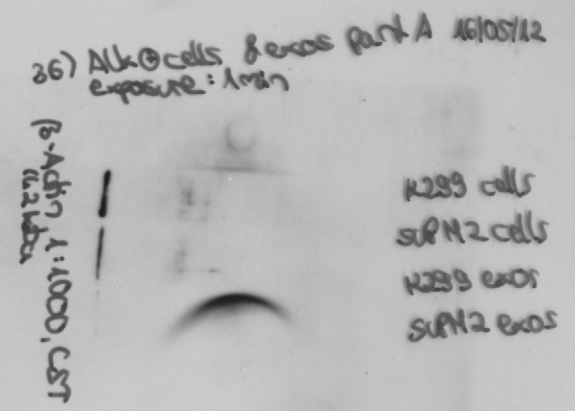
36) Alk⊖cells & exos part A 16/05/12
exposure: 1min
H299 cells
SuPM2 cells
H299 exos
SuPM2 exos
β-Actin 1:1000, CST
(42 kDa

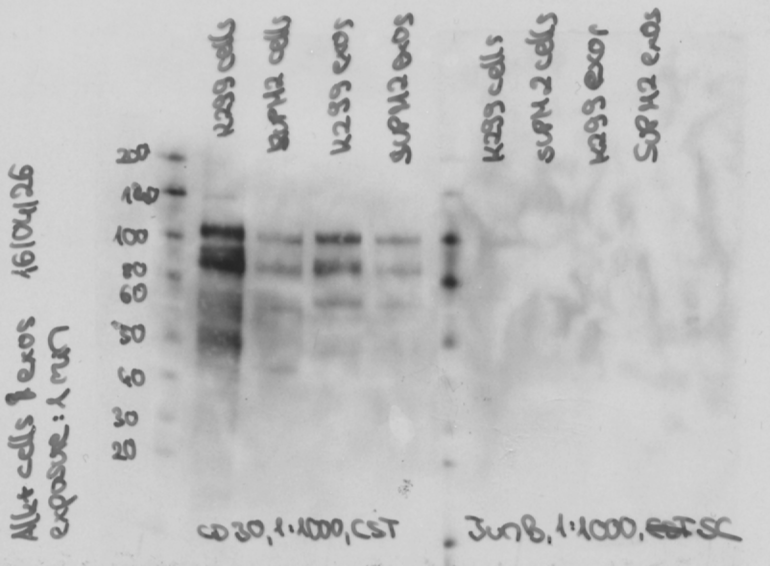

K299 cells
SUPM2 cells
K299 exos
SUPM2 exos
K299 cells
SUPM2 cells
K299 exos
SUPM2 exos
exposure: 1 min
CD30, 1:1000, CST
JunB, 1:1000, SC

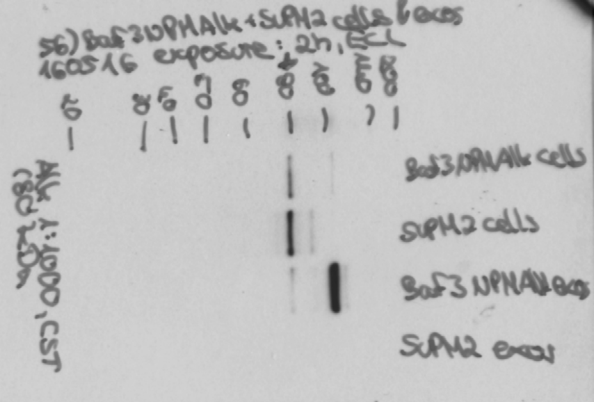
56) Baf3 NPMAlk + SUPM2 cells & exos
160516 exposure: 2h, ECL
Baf3/NPMAlk cells
SUPM2 cells
Baf3 NPMAlk exos
SUPM2 exos
Alk 1:1000, CST
(80 kDa)

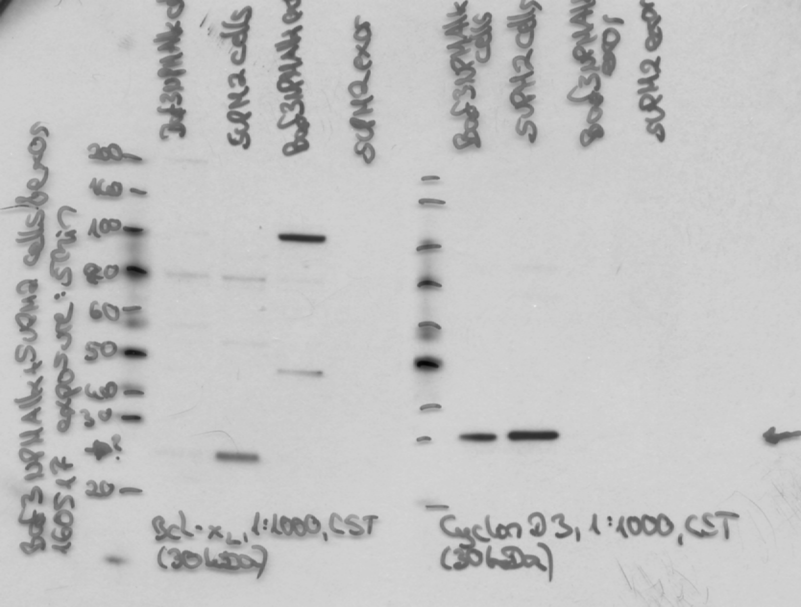

SUDHL2 cells
SUDHL2 exos
Baf3 NPM-ALK cells
Baf3 NPM-ALK exos
250
150
100
80
60
50
40
30
20
Bcl-xL, 1:1000, CST
(30 kDa)
Cyclin D3, 1:1000, CST
(30 kDa)

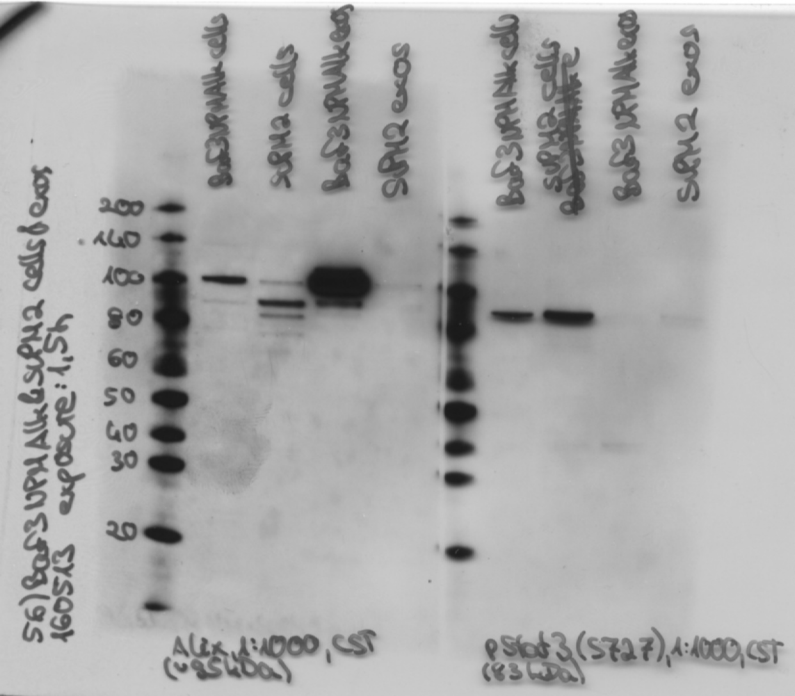
56) BaF3 NPM Alk & SUPM2 cells & exos
160513 exposure: 1,5h
BaF3 NPM Alk cells
SUPM2 cells
BaF3 NPM Alk exos
SUPM2 exos
BaF3 NPM Alk cells
SUPM2 cells
BaF3 NPM Alk exos
SUPM2 exos
200
140
100
80
60
50
40
30
20
Alix, 1:1000, CST
(~95 kDa)
pStat3 (S727), 1:1000, CST
(83 kDa)

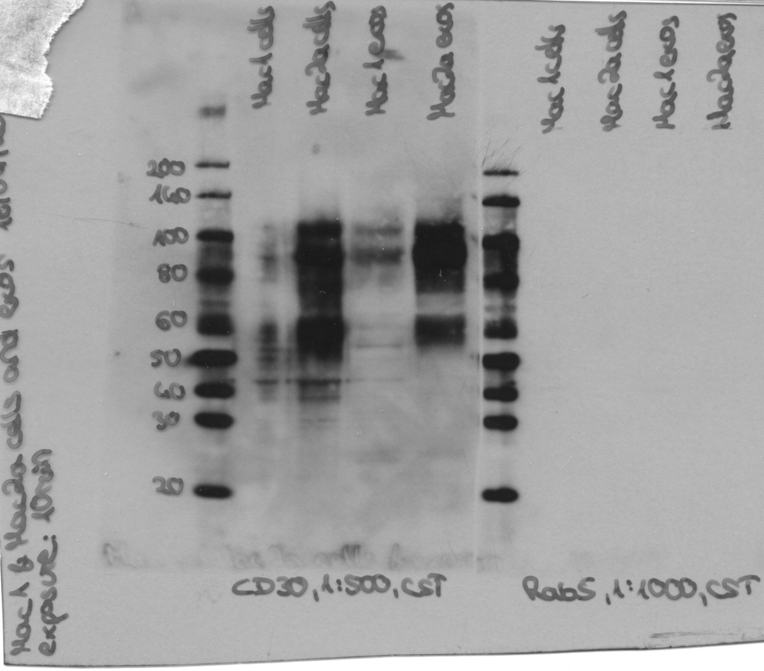
Mac1 cells
Mac2a cells
Mac1 exos
Mac2a exos
Mac1 cells
Mac2a cells
Mac1 exos
Mac2a exos
200
100
80
60
50
40
30
20
CD30, 1:500, CST
Rab5, 1:1000, CST
Mac1 & Mac2a cells and exos
exposure: 10min

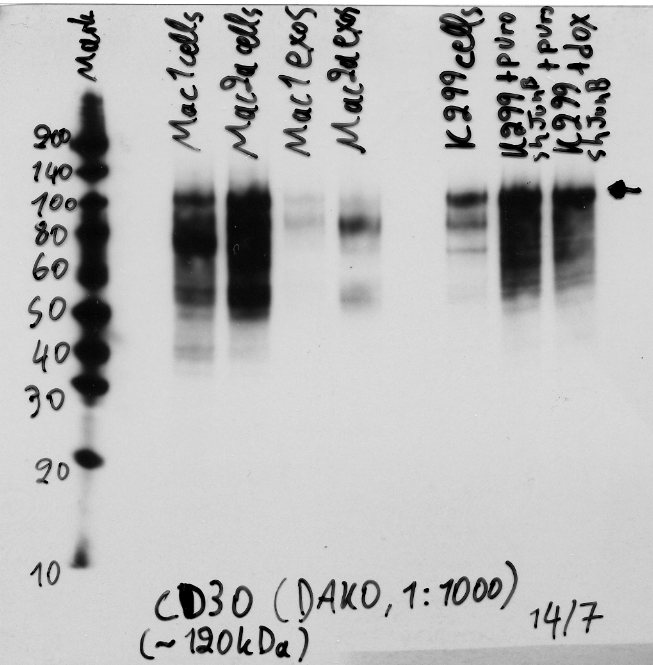
Mark
Mac1 cells
Mac2a cells
Mac1 exos
Mac2a exos
K299 cells
K299 +puro shJunB
+puro
K299 +dox shJunB
200
140
100
80
60
50
40
30
20
10
CD30 (DAKO, 1:1000)
(~120kDa)
14/7

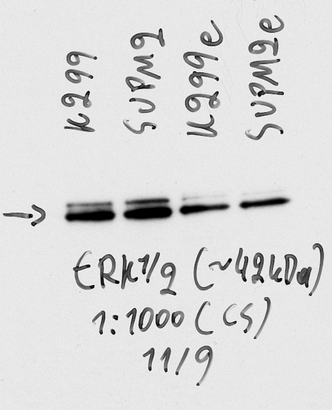
K299
K299e
ERK1/2 (~42kDa)
1:1000 (CS)
11/9

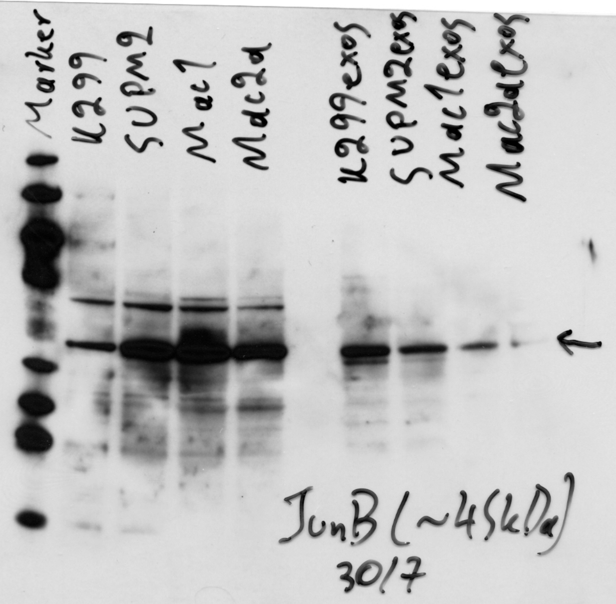
Marker
K299
SUPM2
Mac1
Mac2a
K299exos
SUPM2exos
Mac1exos
Mac2aExos
JunB (~45kDa)
30/7

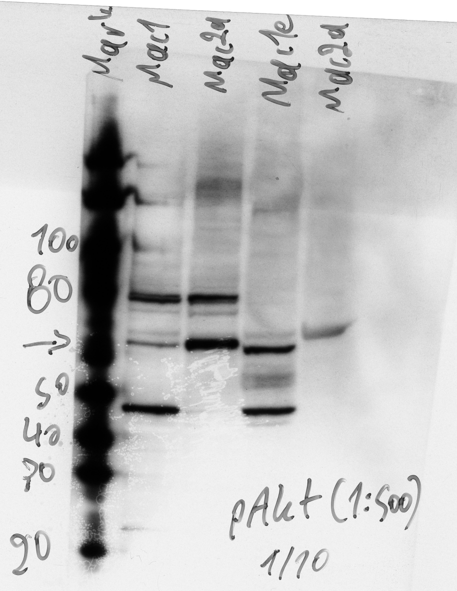

Mac1
100
80
50
40
pAkt (1:500)
1/10

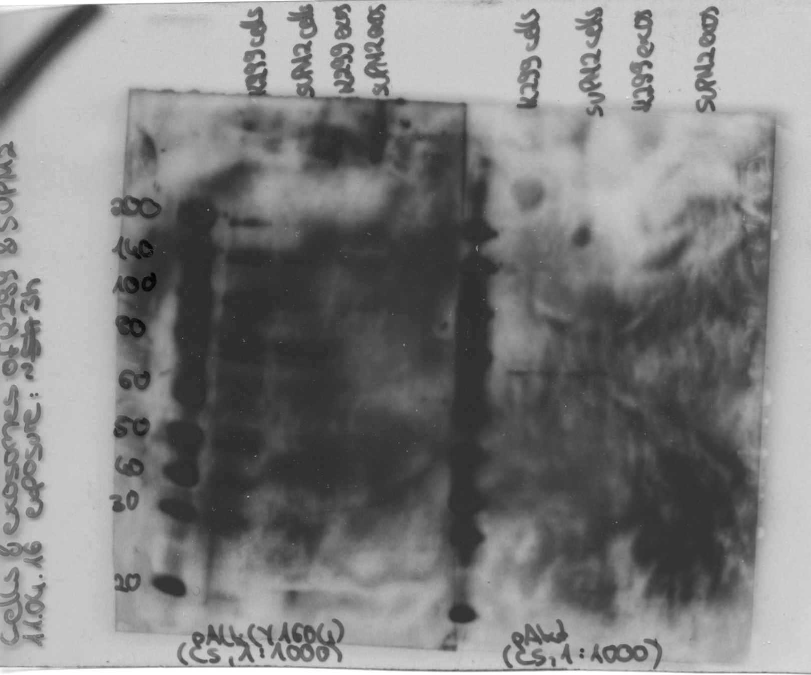

pALK(Y1604)
(CS, 1:1000)
pAkt
(CS, 1:1000)
200
150
100
80
60
50
40
30
20

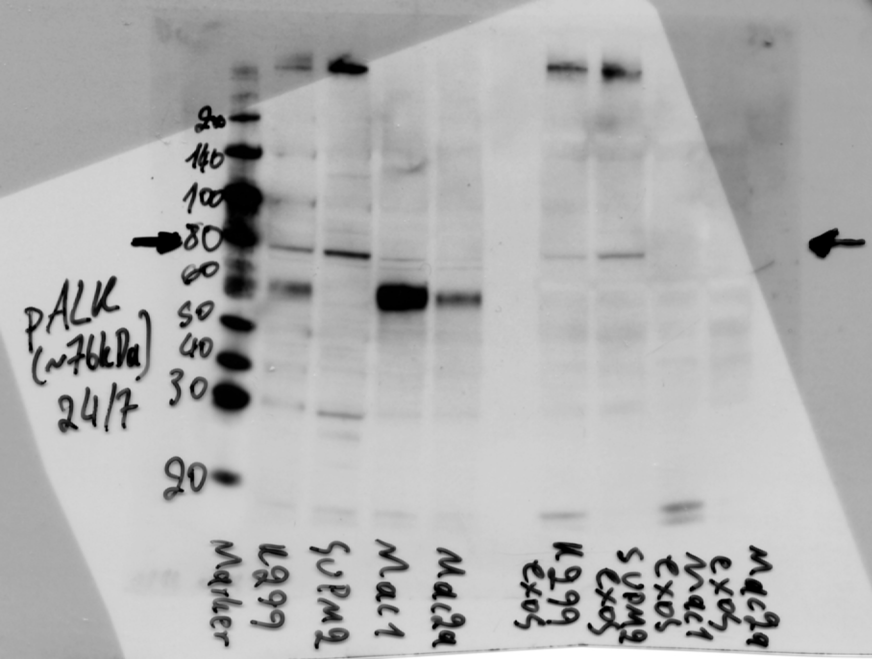
pALK
(~76kDa)
24/7
140
100
80
60
50
40
30
20
Marker
K299
SUPM2
Mac1
Mac2a
K299 exos
SUPM2 exos
exos Mac1
exos Mac2a

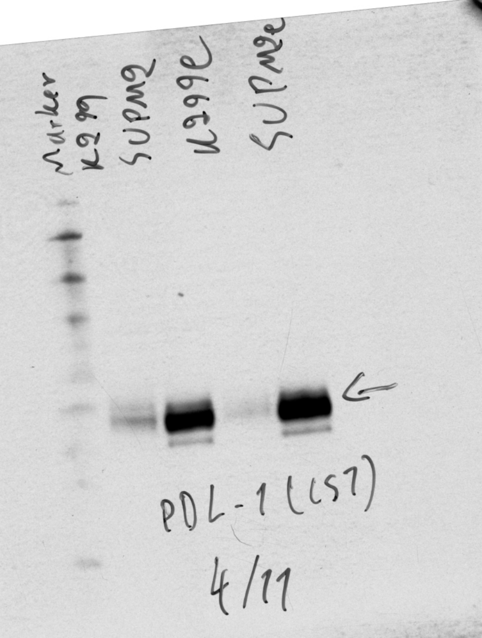
Marker
K299
SUPM2
K299e
SUPM2e
PDL-1 (CST)
4/11

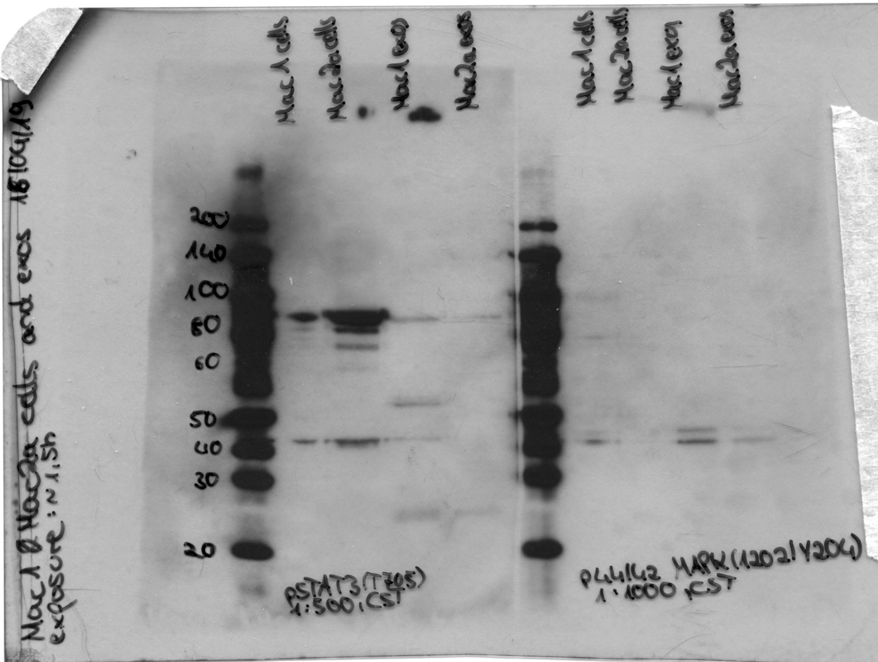
Mac1 & Mac2a cells and exos 18/04/19
exposure: ~1.5h
Mac1 cells
Mac2a cells
Mac1 exos
Mac2a exos
Mac1 cells
Mac2a cells
Mac1 exos
Mac2a exos
200
140
100
80
60
50
40
30
20
pSTAT3 (T705)
1:500, CST
p44/42 MAPK (T202/Y204)
1:1000, CST

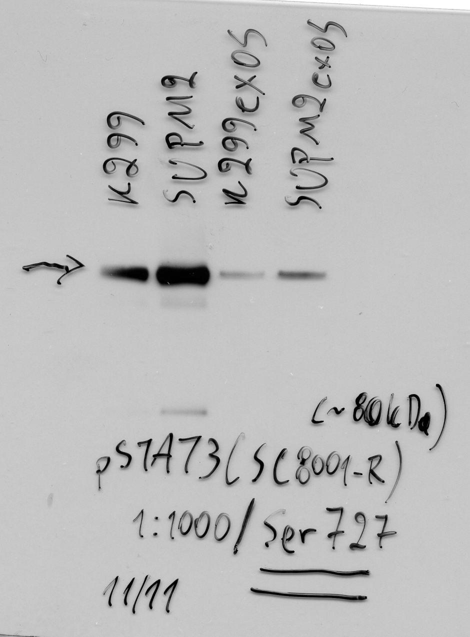
K299
SUPM2
K299cx05
SUPM2cx05
(~80kDa)
pSTAT3 (SC8001-R)
1:1000/Ser727
11/11

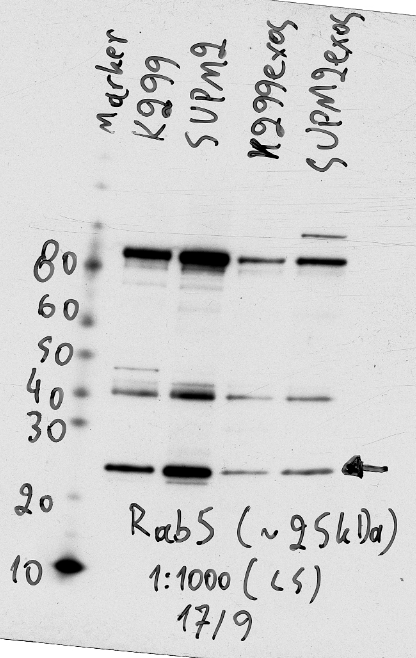

Marker
K999
SUPM2
K999exos
SUPM2exos
80
60
50
40
30
20
10
Rab5 (~25kDa)
1:1000 (LS)
17/9

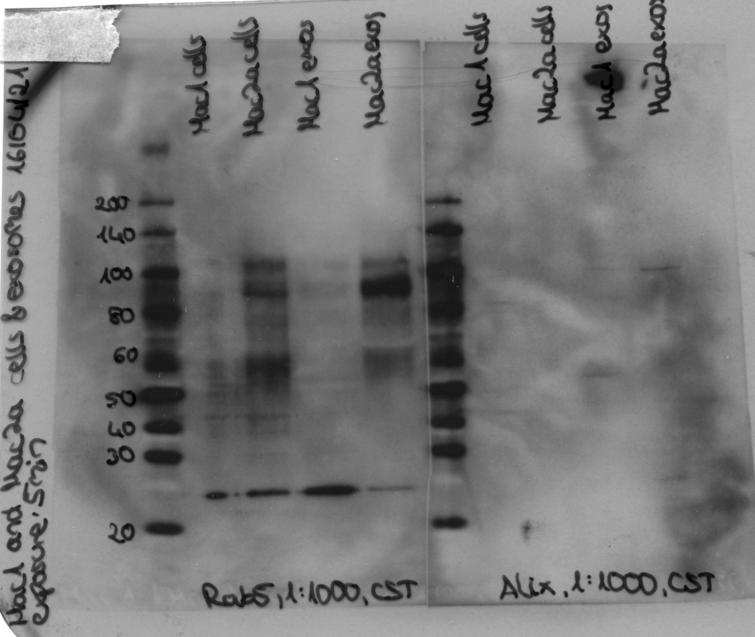

Mac1 and Mac2a cells & exosomes
exposure: 5min
Mac1 cells
Mac2a cells
Mac1 exos
Mac2a exos
200
140
100
80
60
50
40
30
20
Rab5, 1:1000, CST
Mac1 cells
Mac2a cells
Mac1 exos
Mac2a exos
Alix, 1:1000, CST

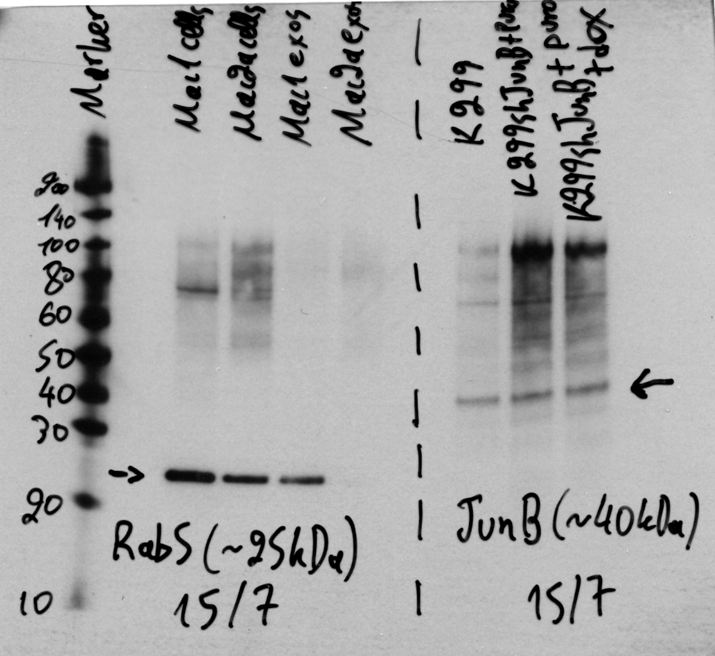

Marker
Mac1 cells
Mac2a cells
Mac1 exos
K299
K299shJunB+puro
K299shJunB+puro
+dox
200
140
100
80
60
50
40
30
20
10
Rab5 (~25kDa)
15/7
JunB (~40kDa)
15/7

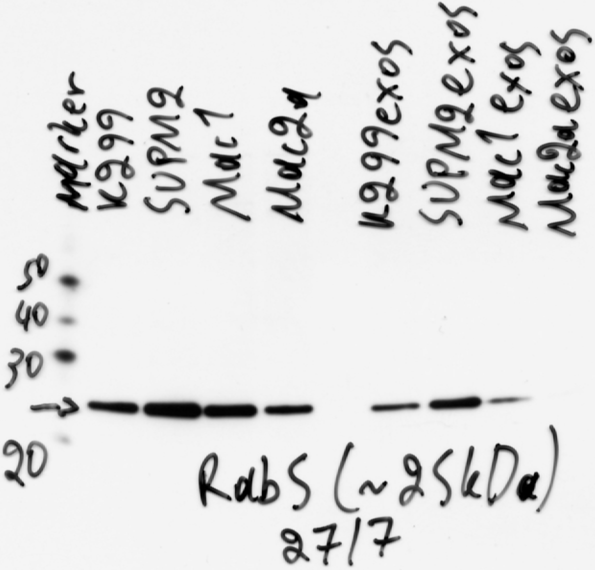

Marker
K299
SUPM2
Mac1
Mac2a
K299exos
SUPM2exos
Mac1exos
Mac2aexos
50
40
30
20
Rab5 (~25kDa)
27/7

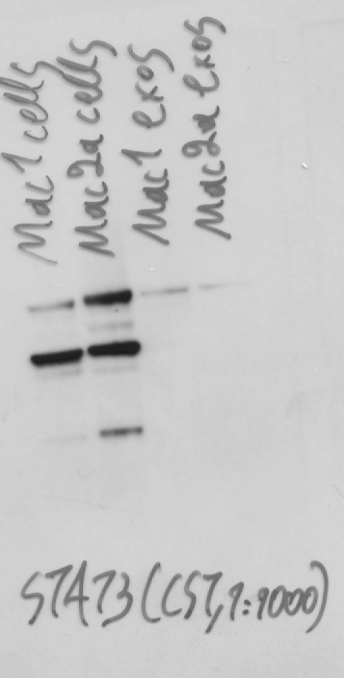
Mac1 cells
Mac2a cells
Mac1 exos
Mac2a exos
STAT3 (C57,1:1000)

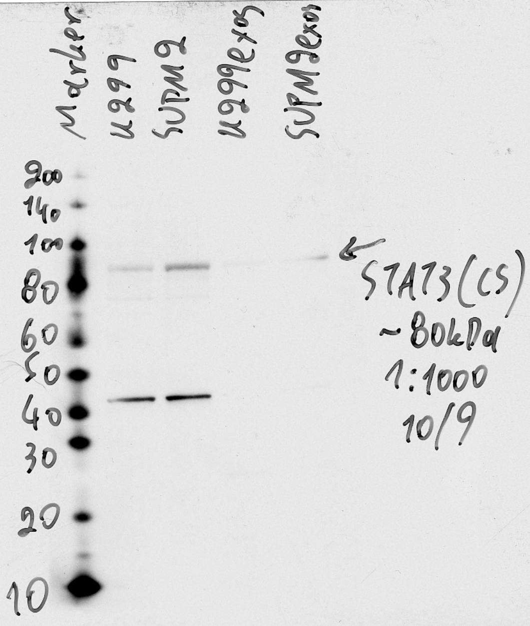
Marker
U299
SUPM2
U299exos
SUPM2exos
200
140
100
80
60
50
40
30
20
10
STAT3 (CS)
~80kDa
1:1000
10/9

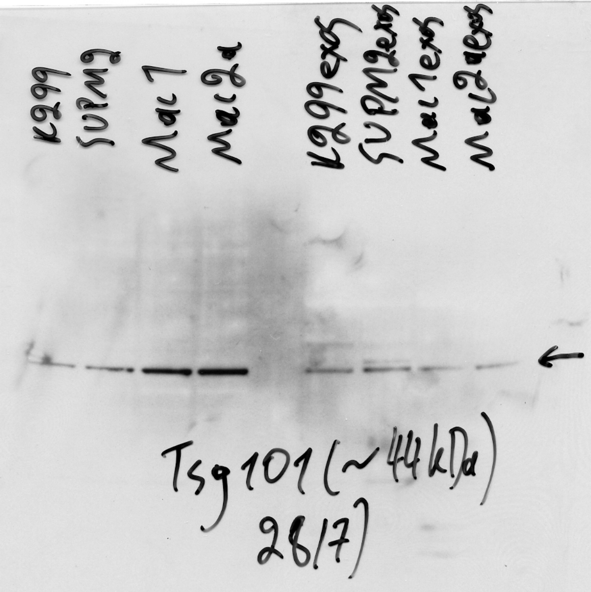
K299
SUPM2
Mac1
Mac2a
K299exos
SUPM2exos
Mac1exos
Mac2aexos
Tsg101 (~44kDa)
28/7
