# Supplementary material for: ALK+ Anaplastic Large Cell Lymphoma (ALCL)-Derived Exosomes Carry ALK Signaling Proteins and Interact with Tumor Microenvironment
Source: Cancers (Basel). 2022 Jun 14;14(12):2939. doi: 10.3390/cancers14122939 (PMC9221431; doi:10.3390/cancers14122939)

Suppl. Table S1. List of cell lines used in the present study

Cell line	Lymphoma type	ALK status	Species
**Karpas 299 ***	Anaplastic large cell lymphoma	Positive	Human
**SUP-M2** [#]	Anaplastic large cell lymphoma	Positive	Human
**Mac-1 ***	Anaplastic large cell lymphoma	Negative	Human
**Mac-2A ***	Anaplastic large cell lymphoma	Negative	Human
**L88 ****	Bone marrow derived fibroblasts	Negative	Human
**Ba/F3**[#]	Pro-B murine lymphoid cells	Negative	Mouse

Purchased from ATCC, USA
* A gift from Dr. Marshal Kadin (Boston, MA, USA)
# Purchased from DSMZ, Germany
****** The L88 bone marrow stromal cells were kindly provided by Dr. Chiara Corsini (European Institute of Oncology, Milan, Italy)

SUPPL. FIGURE S1: Donsitometry data for Western blots are shown below for all relevant figures

Figure 1c

ALK+ ALCL

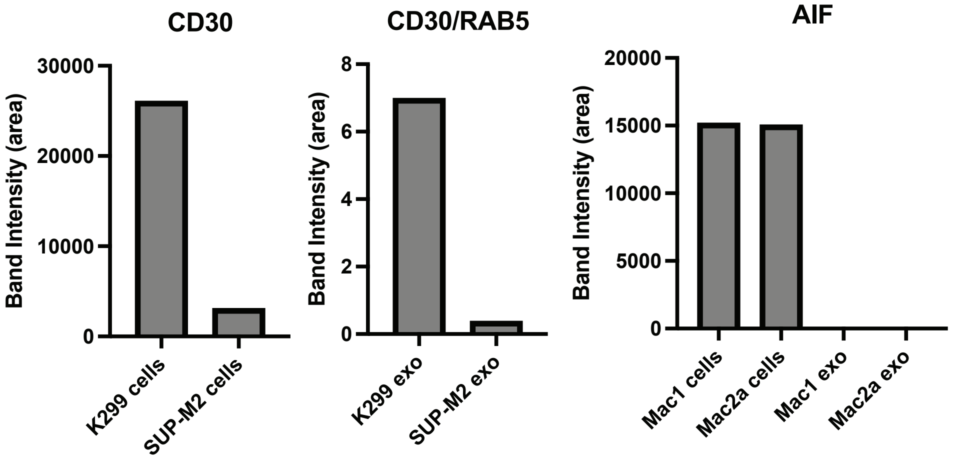

ALK- ALCL

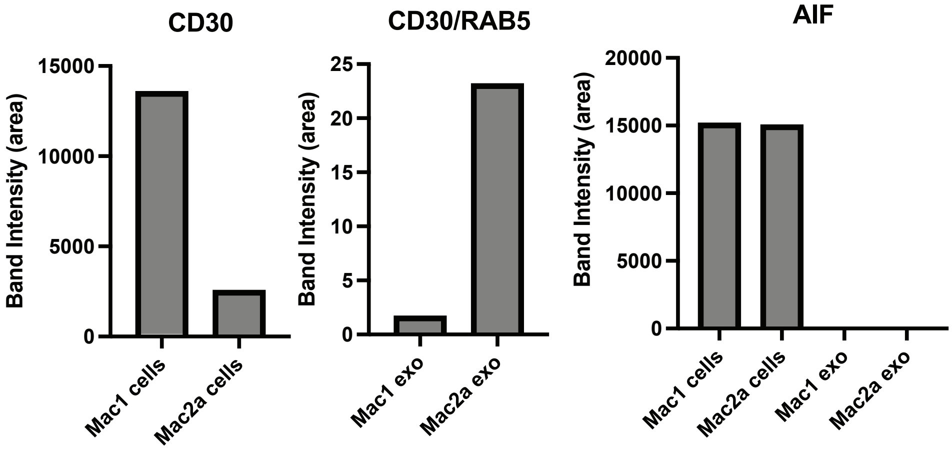

Figure 1d ALK+

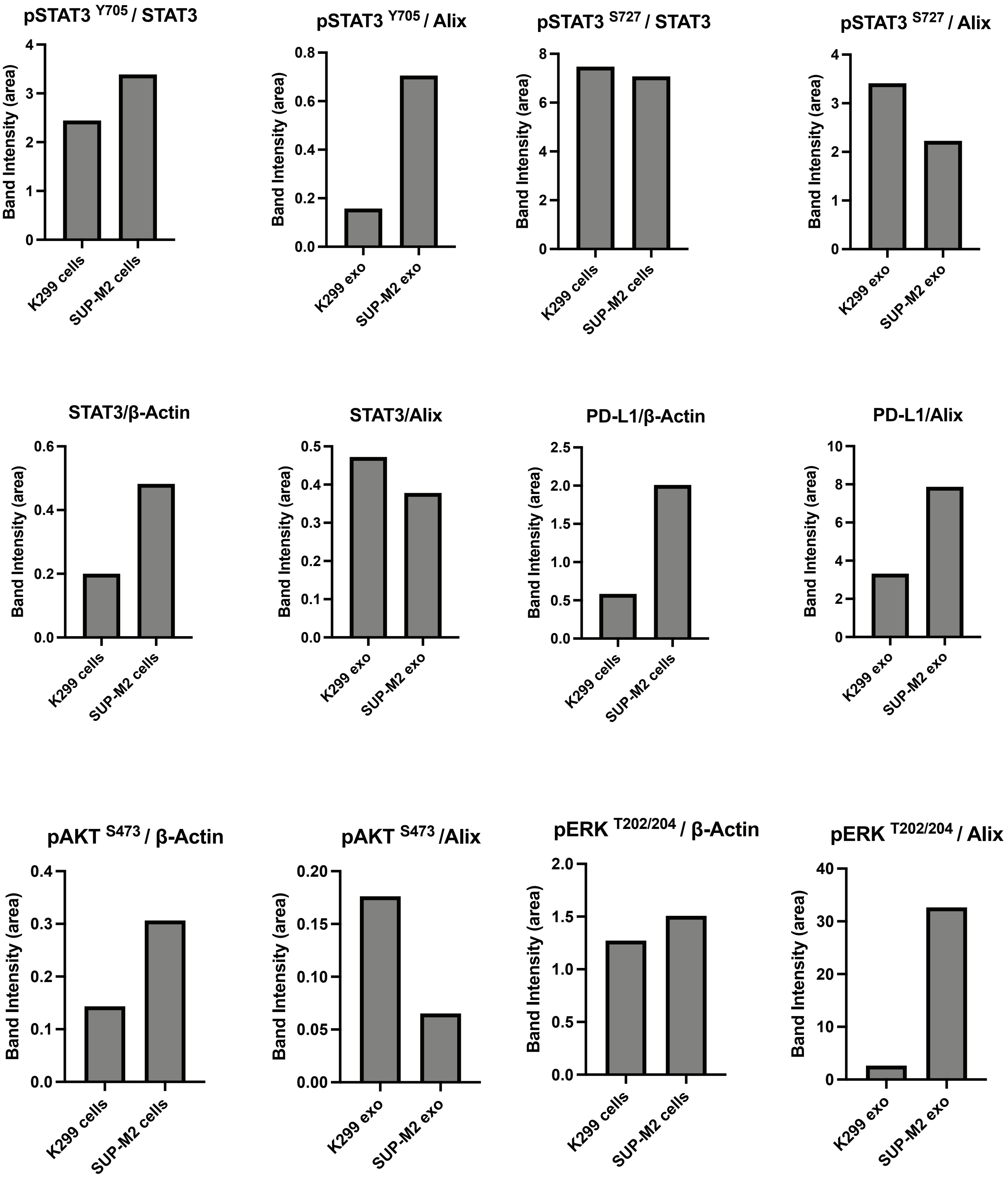

Figure 1d ALK-

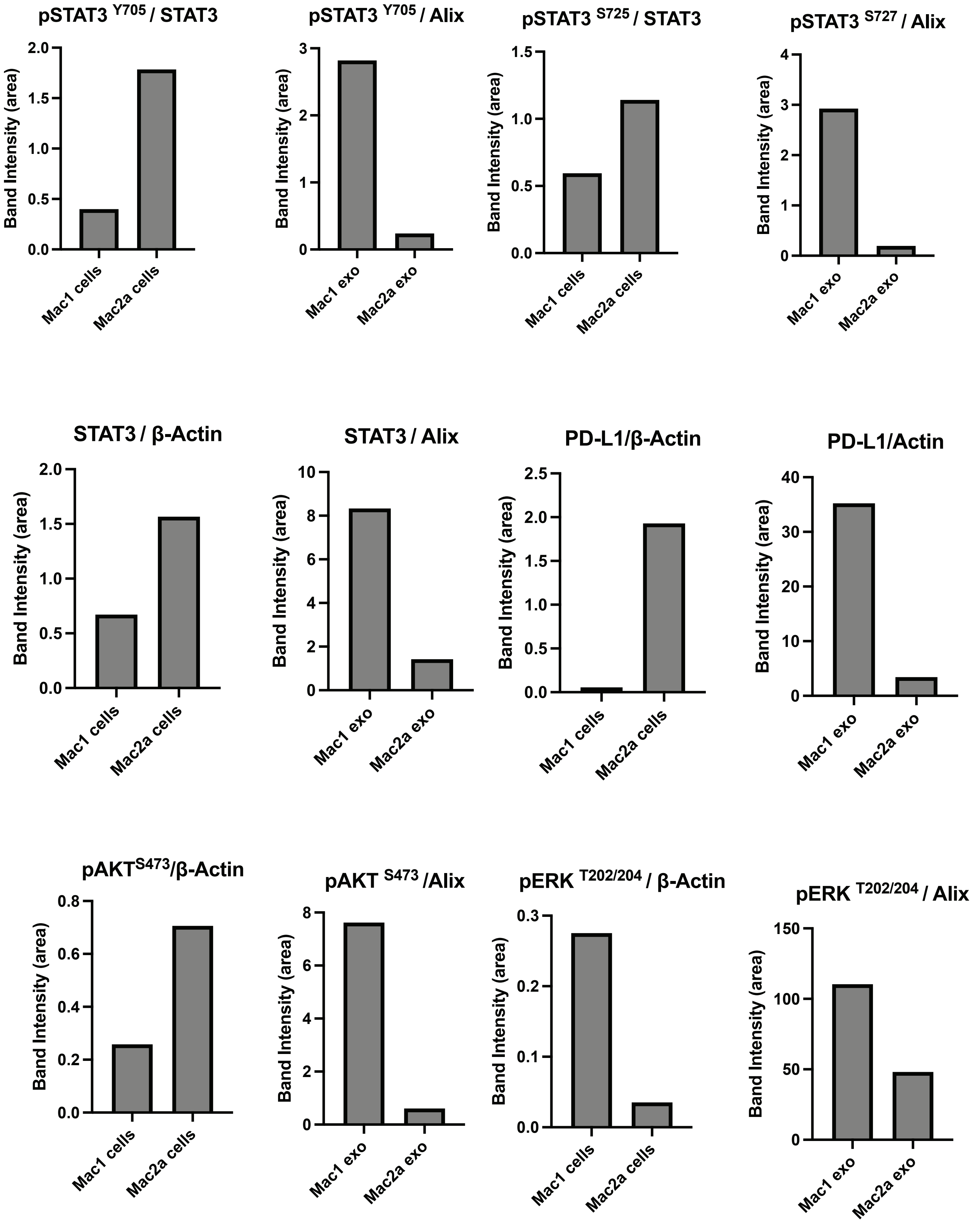

Figure 1E

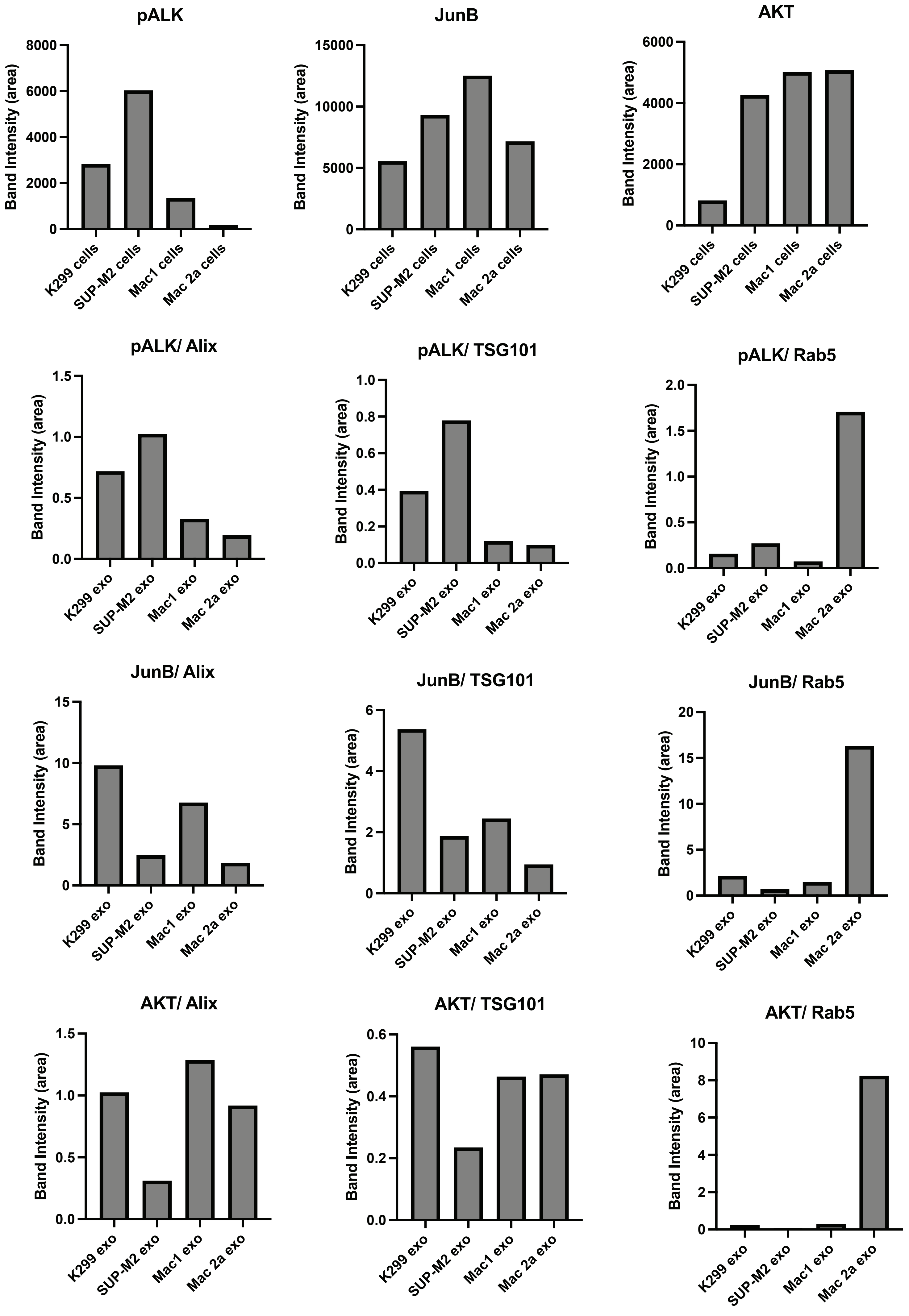
pALK
Band Intensity (area)
0
2000
4000
6000
8000
K299 cells
SUP-M2 cells
Mac1 cells
Mac 2a cells
JunB
Band Intensity (area)
0
5000
10000
15000
K299 cells
SUP-M2 cells
Mac1 cells
Mac 2a cells
AKT
Band Intensity (area)
0
2000
4000
6000
K299 cells
SUP-M2 cells
Mac1 cells
Mac 2a cells
pALK/ Alix
Band Intensity (area)
0.0
0.5
1.0
1.5
K299 exo
SUP-M2 exo
Mac1 exo
Mac 2a exo
pALK/ TSG101
Band Intensity (area)
0.0
0.2
0.4
0.6
0.8
1.0
K299 exo
SUP-M2 exo
Mac1 exo
Mac 2a exo
pALK/ Rab5
Band Intensity (area)
0.0
0.5
1.0
1.5
2.0
K299 exo
SUP-M2 exo
Mac1 exo
Mac 2a exo
JunB/ Alix
Band Intensity (area)
0
5
10
15
K299 exo
SUP-M2 exo
Mac1 exo
Mac 2a exo
JunB/ TSG101
Band Intensity (area)
0
2
4
6
K299 exo
SUP-M2 exo
Mac1 exo
Mac 2a exo
JunB/ Rab5
Band Intensity (area)
0
5
10
15
20
K299 exo
SUP-M2 exo
Mac1 exo
Mac 2a exo
AKT/ Alix
Band Intensity (area)
0.0
0.5
1.0
1.5
K299 exo
SUP-M2 exo
Mac1 exo
Mac 2a exo
AKT/ TSG101
Band Intensity (area)
0.0
0.2
0.4
0.6
K299 exo
SUP-M2 exo
Mac1 exo
Mac 2a exo
AKT/ Rab5
Band Intensity (area)
0
2
4
6
8
10
K299 exo
SUP-M2 exo
Mac1 exo
Mac 2a exo

Figure 1F

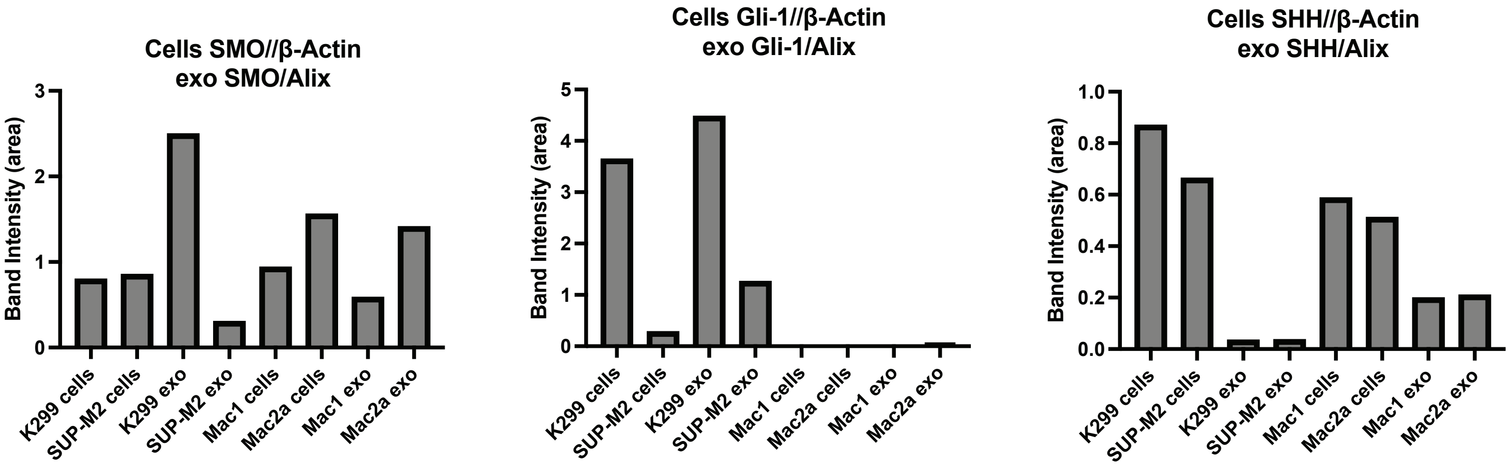

Figure 2C

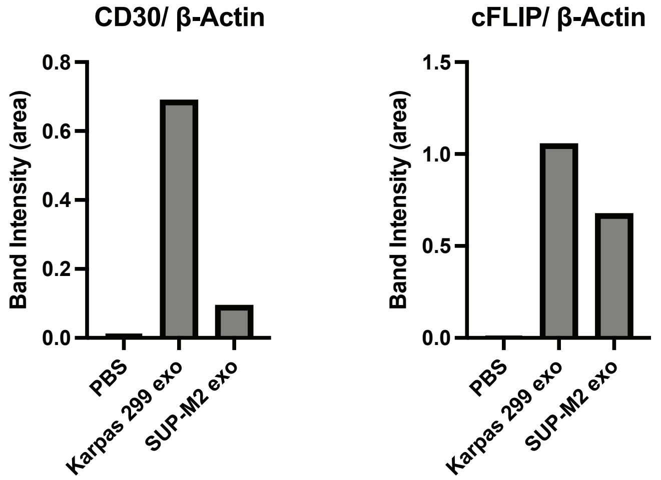

Figure 2F

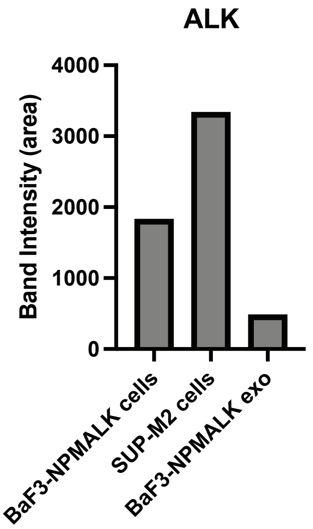

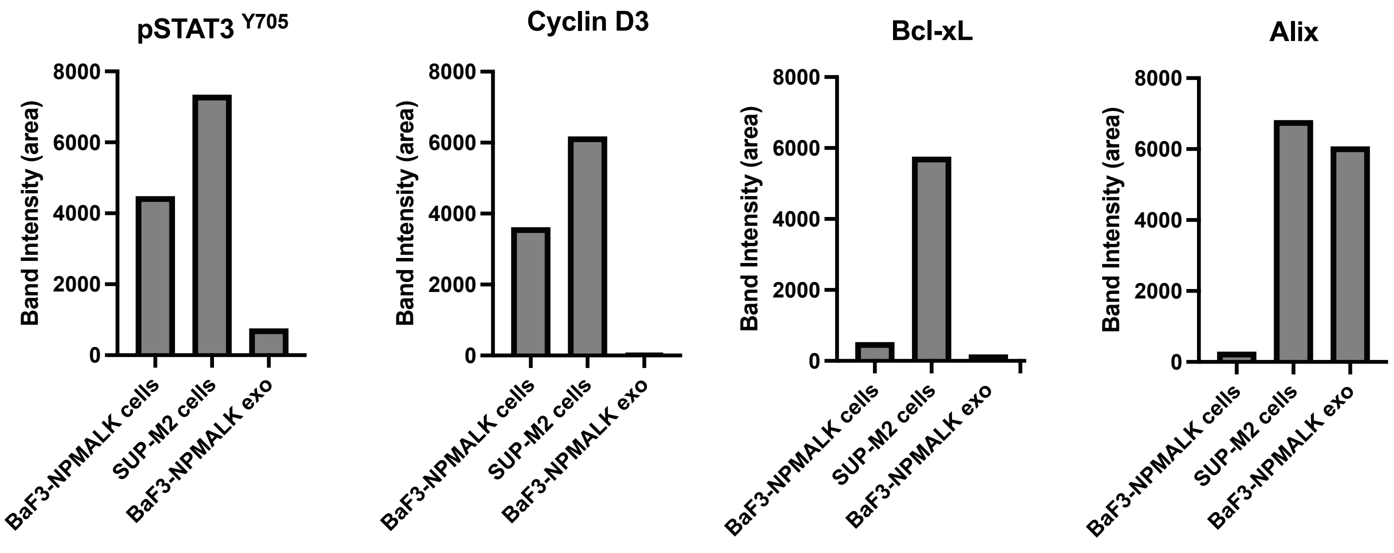

Supplement: Supplementary file 1 [file cancers-14-02939-s001.zip › cancers-1672707-tableS1 and figureS1.pdf]
